# CSF *H3F3A* K27M circulating tumor DNA copy number quantifies tumor growth and in vitro treatment response

**DOI:** 10.1186/s40478-018-0580-7

**Published:** 2018-08-15

**Authors:** Stefanie Stallard, Masha G. Savelieff, Kyle Wierzbicki, Brendan Mullan, Zachary Miklja, Amy Bruzek, Taylor Garcia, Ruby Siada, Bailey Anderson, Benjamin H. Singer, Rintaro Hashizume, Angel M. Carcaboso, Kaitlin Q. McMurray, Jason Heth, Karin Muraszko, Patricia L. Robertson, Rajen Mody, Sriram Venneti, Hugh Garton, Carl Koschmann

**Affiliations:** 1 Department of Pediatrics, Michigan Medicine, University of Michigan Medical School, 3520D MSRB I, 1150 W Medical Center Drive, Ann Arbor, MI 48109 USA; 2 SciGency Science Communications, Ann Arbor, MI 48104 USA; 3 Department of Neurosurgery, Michigan Medicine, University of Michigan, Ann Arbor, MI 48109 USA; 4 Department of Internal Medicine, Michigan Medicine, University of Michigan, Ann Arbor, MI 48109 USA; 5 Department of Neurological Surgery, Feinberg School of Medicine, Northwestern University, Chicago, IL 60611 USA; 6 Department of Biochemistry and Molecular Genetics, Feinberg School of Medicine, Northwestern University, Chicago, IL 60611 USA; 7 Department of Oncology, Hospital Sant Joan de Déu, 08950 Barcelona, Spain; 8 Department of Pathology, Michigan Medicine, University of Michigan, Ann Arbor, MI 48109 USA

Diffuse intrinsic pontine glioma (DIPG) is a lethal childhood brain cancer and patients face a grim prognosis with few treatment options [7]. Targeted therapies based on actionable genetic mutations may offer DIPG patients novel treatment regimens [9, 10]. Although whole exome sequencing (WES) of tumor tissue can fully characterize the somatic mutational profile, it requires a surgical procedure and is relatively costly and time consuming. Consequently, less invasive and more rapid diagnostic tests are needed to detect actionable brain cancer mutations.

Brain tumors and metastases to the brain shed circulating tumor DNA (ctDNA) into the cerebrospinal fluid (CSF), which can be leveraged for the detection of tumor-associated genetic mutations from minimally invasive lumbar punctures [16]. Droplet digital PCR (ddPCR) is an ultrasensitive PCR method that can detect low copy numbers of DNA, including ctDNA, in CSF [13]. It has proven adept for the detection of ctDNA mutations in CSF from patients with primary brain tumors [3, 5, 14] and central nervous system (CNS) metastases from other cancers [3, 8, 12, 14, 15, 17].

The majority of DIPGs possess a recurrent, potentially actionable mutation to histone 3 (either *H3F3A* or *HIST1H3B*) at lysine position 27 (K27M). H3K27M detection in CSF by a combination of nested PCR and Sanger sequencing in DIPG patients [6] as well as by ddPCR in older diffuse midline glioma patients has been reported [11]. Thus far, there have been no extensive studies using ddPCR to quantify ctDNA in the CSF of younger pediatric DIPG patients. Additionally, there are significant gaps in our knowledge, including whether ctDNA abundance depends on location of sample collection and whether ctDNA can quantify tumor growth and treatment response. To help answer these questions, we developed a novel ddPCR assay for the *H3F3A* K27M mutation and applied it to four pediatric patients with *H3F3A* K27M-mutant DIPG and non-brainstem GBM, including multi-focal sampling of one patient. Additionally, we generated an in vitro co-culture model of DIPG cells and astrocytes (NHA), evaluating their release of DNA into cell culture media as a means to simulate ctDNA release into the CSF.

We employed ddPCR because it is a rapid, simple, and ultra-sensitive method of DNA detection capable of accurate quantification down to very low copy number [13]. We designed PCR probes specific to wild-type (WT) *H3F3A* and mutant K27M sequences (Additional file 1: supporting information, SI), which were validated for low copy detection and linearity by serial dilution of synthetic K27M mutant sequence oligonucleotide (Additional file 2: Figure S1), as well as in control CSF (no CNS tumor) with and without synthetic K27M oligonucleotide (Additional file 2: Figure S2). This fully validated ddPCR method was then used on experimental samples (Fig. 1a). In a prospective cohort of patients who were enrolled in the IRB-approved University of Michigan Brain Tumor CSF Registry, CSF ddPCR results were compared to contrast-enhancing and total tumor cross-sectional area on MRI.

**Fig. 1:**
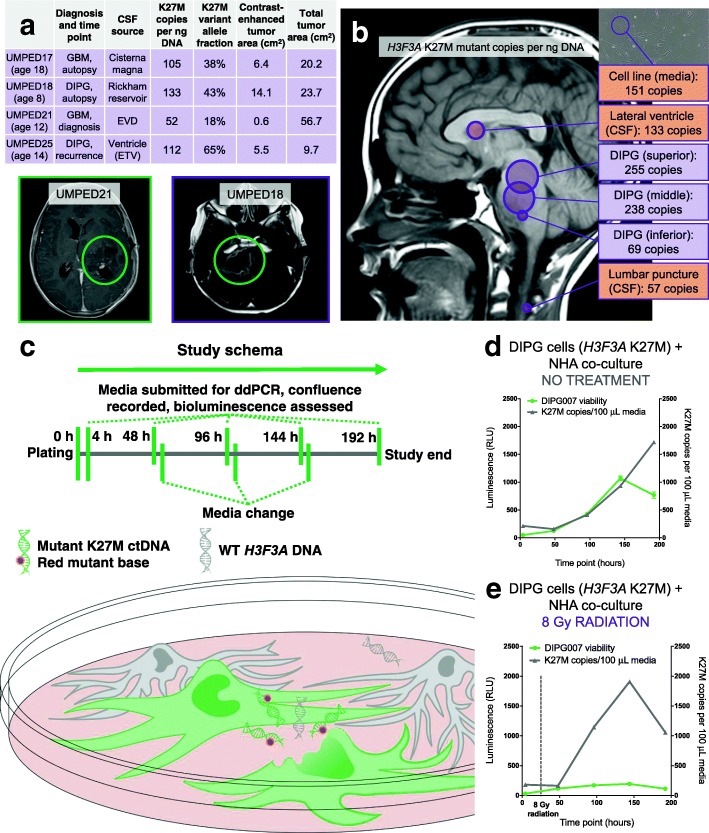
**a** CSF ddPCR results from experimental samples correlated with contrast-enhancing and total tumor cross-sectional area on MRI. **b** ddPCR of multi-focal sampling shows K27M copy number varies between tumor (purple) and CSF (orange) regions **c** Co-culture scheme of bioluminescent human DIPG007 cells with NHAs. **d** DIPG007 cells release ctDNA in proportion to their proliferation. **e** 8 Gy radiation results in an increase in mutant ctDNA from DIPG007 cells

We found that ddPCR was able to detect the K27M mutation in patient CSF and that the closest relationship emerged between mutant K27M copies per ng of total DNA (hereafter K27M copies) and contrast-enhancing cross-sectional tumor area on MRI (Fig. 1a). We then used ddPCR for multi-focal sampling of an eight-year-old patient with DIPG at autopsy (UMPED18) and observed that K27M copies varied throughout the tumor (Fig. 1b). The number of K27M copies was two-fold higher in CSF from the lateral ventricle as compared to CSF from a lumbar puncture, in accordance with prior studies that have suggested that ctDNA release into the CSF may be reliant upon the location of the tumor adjacent to a CSF reservoir [16]. If this finding is confirmed in future cases with multi-focal sampling, lumbar samples may have reduced sensitivity for CSF ctDNA compared to ventricular samples.

To better understand changes in K27M copy number in response to both growth and treatment of DIPG cells, we created an experimental in vitro model of bioluminescent human DIPG007 cells co-cultured with NHAs (Fig. 1c). We found that DIPG007 cells released more ctDNA into culture media in proportion to their proliferation (Fig. 1d), even when the media was changed frequently to approximate the constant production and resorption of CSF. This suggests that, at least in part, ctDNA correlates with tumor cell proliferation. However, irradiation with 8 Gy resulted in a dramatic increase in mutant ctDNA approximately 72–120 h post radiotherapy (Fig. 1e) before tapering off. The results suggest ddPCR may be a viable method for monitoring response to therapy with an early release of ctDNA indicative of an effective treatment.

The dawn of precision medicine, and its potential benefit to patients, has spurred research into faster, simpler, and less invasive methods of detection of actionable tumor-associated mutations. Due to its great sensitivity and low limit of detection, ddPCR has been used to detect tumor mutations in CSF from a range of cancer patients [1–5, 8, 12, 14, 15, 17]. However, there has been little elaboration within the literature on whether tumor size may be related to the amount of ctDNA detected by ddPCR or its suitability to track response to treatment.

Our pilot study suggests that *H3F3A* K27M copies in the CSF of children with DIPG and high-grade glioma have a linear relationship with contrast-enhancing cross-sectional tumor area and confirms the importance of proximity of a sample to the tumor. The former observation was further supported by in vitro experiments showing that tumor cell proliferation results in increased ctDNA and that *H3F3A* K27M copies can be used to follow treatment response due to temporarily enhanced ctDNA release shortly after effective therapies. Our study lays the ground work for the inclusion of CSF analysis with surveillance MRIs in the treatment of this patient population.

## Additional files

Additional file 1: **Supplemental Information.** Detailed methods and H3F3A K27M assay design. (DOCX 28 kb)

Additional file 2: **Figure S1.** Serial dilution of K27M mutant oligonucleotide in constant background of wild-type DNA demonstrates consistent detection down to at least 2% VAF under typical experimental conditions, with the possibility of detection at even lower VAF under ideal conditions. One such dilution series is shown above, with **(a)** showing number of droplets positive for mutant or wild-type *H3F3A* sequence and **(b)** showing the corresponding VAF values. **Figure S2.** Plot of droplets (blue – positive mutant *H3F3A* K27M, green – positive wildtype *H3F3A*, grey – negative droplets) from ddPCR performed on **(a)** non-tumor human CSF spiked with synthetic K27M mutant sequence oligonucleotide and **(b)** non-tumor human CSF alone. (DOCX 268 kb)

## Abbreviations

CNS: Central nervous system
CSF: Cerebrospinal fluid
ctDNA: Circulating tumor DNA
ddPCR: Droplet digital PCR
DIPG: Diffuse intrinsic pontine glioma
GBM: Glioblastoma
LOD: Limit of detection
NHA: Normal human astrocyte
WES: Whole exome sequencing
WT: Wild-type
