# Supplementary material for: CSF H3F3A K27M circulating tumor DNA copy number quantifies tumor growth and in vitro treatment response
Source: Acta Neuropathol Commun. 2018 Aug 15;6:80. doi: 10.1186/s40478-018-0580-7 (PMC6094898; doi:10.1186/s40478-018-0580-7)
Supplement: Supplementary file 1 — Supplemental Information. Detailed methods and H3F3A K27M assay design. (DOCX 28 kb) [file 40478_2018_580_MOESM1_ESM.docx]

**Patient samples**

Informed consent/assent was obtained according to University of Michigan policy and the IRB-approved Brain Tumor CSF Registry (HUM00132228). All patient samples were obtained at the time of a clinically-indicated surgical intervention, lumbar puncture, or autopsy. Non-tumor CSF was obtained from patients with normal pressure hydrocephalus undergoing therapeutic CSF drainage (HUM00128299). CSF samples were centrifuged at 1500 rpm for 5 minutes to remove cellular contents and/or debris and supernatant stored at -80 °C until ready for use. Tissue samples were flash frozen and stored at -80 °C until ready for use. Cell culture media was centrifuged as described above and stored at 4 °C for less than 5 days prior to use.

**Magnetic resonance imaging (MRI)**

To determine total cross-sectional tumor area and contrast-enhancing cross-sectional tumor area, MRI images were selected from the imaging study closest to the time of CSF sample collection. Cross-sectional area was determined using the axial image featuring maximal tumor diameter and calculated as the product of this maximal diameter measurement and the diameter perpendicular in the same plane. Measurements of total cross-sectional tumor area were taken from T1-weighted images prior to contrast administration and measurements of contrast-enhancing cross-sectional tumor area after the administration of contrast agent.

**Primary cell line generation**

Human tumor primary cell culture UMPED18 was generated by harvesting tumor cells at the time of autopsy after obtaining University of Michigan autopsy research consent. The tumor tissue was transferred on ice, immediately dissociated using non-enzymatic cell dissociation buffer (Gibco, 13151-014), and then filtered. The cells were maintained in adherent cell line medium comprised of DMEM/F-12 medium (Gibco, 11330-032), 10% fetal bovine serum (VWR, 89510-186), antibiotic-antimycotic (Gibco 15240-062), and normocin (Invivogen, ant-nr-1).

**DNA sequencing**

Whole exome (paired tumor and germline DNA) and transcriptome (tumor RNA) sequencing was performed on all samples according to previously published methodology [1-4] in the Michigan Center for Translational Pathology through PEDS-MIONCOSEQ. The PEDS-MIONCOSEQ study was approved by the University of Michigan Institutional Review Board and adheres to the Clinical Laboratory Improvement Amendments (CLIA).

**DNA isolation**

DNA was isolated from fluid samples using the QIAamp Circulating Nucleic Acid kit (Qiagen, 55114) and from tissue using the DNeasy Blood & Tissue kit (Qiagen, 69506). All concentration measurements were performed using the Qubit 3.0 Fluorometer (Invitrogen, Q33216) and Qubit dsDNA HS Assay kit (Invitrogen, Q32851).

**ddPCR primer design and assay**

Primers for the *H3F3A* K27M assay were designed using Primer3Plus (https://primer3plus.com/cgi-bin/dev/primer3plus.cgi) and probes were designed by a Scientific Applications Specialist at Integrated DNA Technologies (IDT). Locked nucleic acid (LNA) bases were incorporated into the probe design to increase melting temperature without lengthening probe sequence. Both primers and probes were synthesized by IDT.

Forward primer: 5′-GGTAAAGCACCCAGGAAG-3′

Reverse primer: 5′-CAAGAGAGACTTTGTCCC-3′

WT probe with LNA: 5′-HEX-TC+GC+A+A+GA+GT+GC-IABkFQ-3′, “+” denotes LNA bases, HEX: hexachlorofluorescein, IABkFQ: Iowa Black® FQ quencher

K27M probe with LNA: 5′-6-FAM-TC+GC+A+**T**+GA+GTGC-IABkFQ-3′, mutant base is bold, “+” denotes LNA bases, 6-FAM: 6-carboxyfluorescein, IABkFQ: Iowa Black® FQ quencher

Reaction mixtures were prepared using ddPCR Supermix for Probes (no dUTP) (Bio-Rad, 1863024). The following PCR protocol was used: 1 cycle at 95 °C for 10 minutes, 40 cycles at 94 °C for 30 seconds and 58 °C for 1 minute, 1 cycle at 98 °C for 10 minutes, and 1 cycle at 12 °C infinite, all at a ramp rate of 2 °C/second.

**DNA preamplification**

An initial preamplification reaction was occasionally run prior to ddPCR in the case of very low DNA concentration. This was done using the same primers described above and the Q5 High-Fidelity 2X Master Mix (New England BioLabs, M0492S). The following PCR protocol was used: 1 cycle at 98 °C for 30 seconds, 12 cycles at 98 °C for 10 seconds, 58 °C for 30 seconds, and 72 °C for 30 seconds, 1 cycle at 72 °C for 2 minutes, and 1 cycle at 12 °C infinite.

**ddPCR analysis**

The Bio-Rad QX200 AutoDG system was used for all ddPCR work. QuantaSoft Analysis Pro (Bio-Rad) was used for initial analysis of results. The following additional calculations were performed:

Mutant copies/ng total DNA = (MC/DIV) / DIC

Mutant copies/mL CSF or culture media = [(MC/DIV) * DIVT] / OS

MC = number of mutant copies per well

DIV = volume of DNA isolate per well (μL)

DIC = concentration of DNA isolate (ng/μL)

DIVT = volume of total DNA isolate (μL); volume of elution buffer used for DNA isolation

OS = volume of original sample (CSF or culture media) utilized in DNA isolation reaction (mL)

**Cell co-culture**

Normal human astrocytes (henceforth NHA) and HSJD-DIPG-007 (henceforth DIPG007) cells were both grown adherently in TSM base medium with 5% fetal bovine serum (VWR, 89510-186). TSM base was prepared using the following: neurobasal-A medium (Gibco, 10888-022), DMEM/F-12 medium (Gibco, 11330-032), HEPES buffer (Gibco, 15630-080), sodium pyruvate (Gibco, 11360-070), MEM non-essential amino acids (Gibco, 11140-050), GlutaMAX-I supplement (Gibco, 35050-061), antibiotic-antimycotic (Gibco, 15240-096), B-27 supplement minus vitamin A (Gibco, 12587-010), EGF and FGF (Shenandoah Biotech, 100-26 and 100-146), PDGF-A and PDGF-B (Shenandoah Biotech, 100-16 and 100-18), and 0.2% heparin (STEMCELL Technologies, 07980). Cells were lifted using HyClone HyQTase Cell Detachment Reagent (GE Healthcare, SV3003001) and quantified using 0.4% trypan blue solution (Corning, 25-900-CI) and hemacytometer. Cells were seeded in multiple wells of two 24-well plates in the following proportion: 20,000 DIPG007 cells and 30,000 NHA cells. One plate received 8 Gy radiation after 24 hours of growth; the other plate was untreated and served as a control. At each of five time points (4, 48, 96, 144, and 192 hours), cell culture media was collected from a designated well in each plate and used for ddPCR. The cells from these wells were also collected using HyQTase and re-plated in triplicate in a 96-well plate. One aliquot of 100 μg/mL D-luciferin (Gold Biotechnology, LUCK-1G) was added to these wells, incubated for 5 minutes, and then assessed for bioluminescence using the Synergy HTX Multi-Mode Microplate Reader in order to evaluate remaining viable DIPG007 population. Confluence of total cell population was assessed throughout using the IncuCyte ZOOM live-cell analysis system. Culture media was discarded and replenished in each of the remaining wells at every time point with the exceptions of the initial 4-hour time point and the final time point.

1 Koschmann C, Wu Y-M, Kumar-Sinha C, Lonigro R, Vats P, Kasaian K, Cieslik M, Cao X, Anderson B, Frank Ket al (2018) Clinically Integrated Sequencing Alters Therapy in Children and Young Adults With High-Risk Glial Brain Tumors. JCO Precision Oncology: 1-34 Doi 10.1200/po.17.00133

2 Mody RJ, Wu YM, Lonigro RJ, Cao X, Roychowdhury S, Vats P, Frank KM, Prensner JR, Asangani I, Palanisamy Net al (2015) Integrative Clinical Sequencing in the Management of Refractory or Relapsed Cancer in Youth. JAMA 314: 913-925 Doi 10.1001/jama.2015.10080

3 Robinson DR, Wu YM, Vats P, Su F, Lonigro RJ, Cao X, Kalyana-Sundaram S, Wang R, Ning Y, Hodges Let al (2013) Activating ESR1 mutations in hormone-resistant metastatic breast cancer. Nature genetics 45: 1446-1451 Doi 10.1038/ng.2823

4 Wu YM, Su F, Kalyana-Sundaram S, Khazanov N, Ateeq B, Cao X, Lonigro RJ, Vats P, Wang R, Lin SFet al (2013) Identification of targetable FGFR gene fusions in diverse cancers. Cancer discovery 3: 636-647 Doi 10.1158/2159-8290.CD-13-0050
