# Supplementary material for: CSF H3F3A K27M circulating tumor DNA copy number quantifies tumor growth and in vitro treatment response
Source: Acta Neuropathol Commun. 2018 Aug 15;6:80. doi: 10.1186/s40478-018-0580-7 (PMC6094898; doi:10.1186/s40478-018-0580-7)
Supplement: Supplementary file 2 — Figure S1. Serial dilution of K27M mutant oligonucleotide in constant background of wild-type DNA demonstrates consistent detection down to at least 2% VAF under typical experimental conditions, with the possibility of detection at even lower VAF under ideal conditions. One such dilution series is shown above, with (a) showing number of droplets positive for mutant or wild-type H3F3A sequence and (b) showing the corresponding VAF values. Figure S2. Plot of droplets (blue – positive mutant H3F3A K27M, green – positive wildtype H3F3A, grey – negative droplets) from ddPCR performed on (a) non-tumor human CSF spiked with synthetic K27M mutant sequence oligonucleotide and (b) non-tumor human CSF alone. (DOCX 268 kb) [file 40478_2018_580_MOESM2_ESM.docx]

**Additional file 2**

Contents:

Figure S1

Figure S2

**Figure S1**


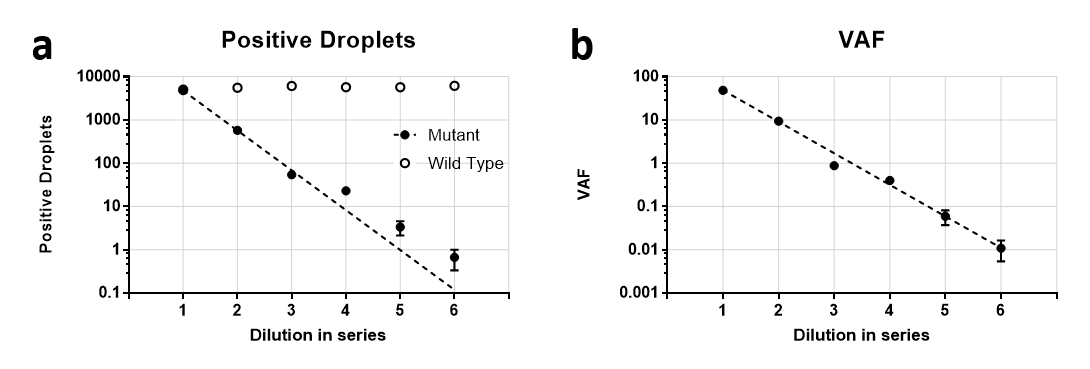


Figure S1: Serial dilution of K27M mutant oligonucleotide in constant background of wild-type DNA demonstrates consistent detection down to at least 2% VAF under typical experimental conditions, with the possibility of detection at even lower VAF under ideal conditions. One such dilution series is shown above, with **(a)** showing number of droplets positive for mutant or wild-type *H3F3A* sequence and **(b)** showing the corresponding VAF values.

**Figure S2**

Figure S2: Plot of droplets (blue – positive mutant *H3F3A* K27M, green – positive wildtype *H3F3A*, grey – negative droplets) from ddPCR performed on **(a)** non-tumor human CSF spiked with synthetic K27M mutant sequence oligonucleotide and **(b)** non-tumor human CSF alone.
